# A study on accuracy and precision of fluid volume measurements by nurses, patients and healthy persons in a clinical setting

**DOI:** 10.1002/nop2.1173

**Published:** 2022-01-05

**Authors:** Charlotte Frydenlund Michelsen, Morten Bo Søndergaard Svendsen, Marie Lommer Bagger, Hanne Konradsen

**Affiliations:** ^1^ SolarSack ApS Copenhagen Denmark; ^2^ Measurelet ApS Kgs Lyngby Denmark; ^3^ Division of Nursing Department of Neurobiology, Care Sciences and Society Karolinska Institute Stockholm Sweden; ^4^ Department of Clinical Medicine Faculty of Health and Medical Sciences University of Copenhagen Copenhagen Denmark; ^5^ Department of Gastroenterology Herlev and Gentofte University Hospital Hellerup Denmark

**Keywords:** accuracy and precision, dehydration, fluid balance, oral rehydration

## Abstract

**Aim:**

To evaluate the accuracy and precision for assessing fluid intake by examining the ability of nurses, patients and healthy people to visually estimate fluid volumes, thereby reflecting the fluid monitoring process in clinical practice.

**Design:**

A cross‐sectional study.

**Methods:**

This study used the convenience sampling method and involved twenty‐five participants from three groups; nurses, patients and healthy people. The participants carried out a set of different visual volume assessments of two types of fluids using two fluid containers. The exact volumes were measured, and the results were compared with the target volumes.

**Results:**

High variations were observed in the fluid volume assessments for patients, nurses and healthy persons and also were observed to be an effect of environmental factors (fluid container or fluid type) on volume perceptions. This highlights the importance of finding new and innovative ways of measuring fluids for oral intake in a hospital setting, to ensure accurate and reliable data on fluid balance and thereby increase patient safety.

## 1 INTRODUCTION

Maintaining a proper fluid balance is essential to ensure functioning of metabolic processes in the body and maintaining health (Armstrong & Johnson, 2018; Diacon & Bell, 2014). The average daily fluid intake varies between individuals. A recent review, which included multiple research studies, found that adults should consume more than 1.8 L of water over 24 hr to maintain adequate hydration (Armstrong & Johnson, 2018). Fluid imbalance results in dehydration or overhydration of patients, with potentially severe consequences. Dehydration is regarded as a loss of fluid resulting in a body mass change of more than 1%. Mild symptoms of dehydration are headache, fatigue and impaired cognitive function. In contrast, prolonged dehydration leads to hypotension, cold hands and feet and weak pulse (Chan et al., 2018; Liska et al., 2019). Overhydration primarily occurs in patients with heart failure or chronic kidney disease, leads to oedema, fatigue and dyspnoea. Failing to identify fluid imbalance symptoms may result in poor recovery, extended hospitalization, complications and ultimately, death (El‐Sharkawy et al., 2015). In a cohort study with 200 hospitalized patients over 65 years of age, 37% were found to be dehydrated at admission, and over two‐third were still dehydrated after 48 hr. The same study showed that the dehydrated patients were six times more likely to die at the hospital than the hydrated patients (El‐Sharkawy et al., 2015). Thus, accurate recordings of fluid balance data play a significant role in understanding and managing a patient's clinical status and for guiding correct treatment (Tattersall, 2016; Vincent & Mahendiran, 2015).

### 1.1 Background

A patient's fluid balance can be measured by assessing and monitoring the input and output of fluids over a specific period of time. However, increasing concerns about inadequate patient hydration from suboptimal monitoring and recording of patients' fluid balance exist across hospitals and healthcare settings globally (Fortes et al., 2015; Jimoh et al., 2015; Johnson et al., 2020; Vincent & Mahendiran, 2015). Several strategies have therefore been suggested to improve the fluid balance recording process, including the use of bed weights, electronic charts, augmented cups and wearable inertial sensors. Still, these interventions are rarely adopted, and patients' fluid balance is typically assessed and monitored manually by the use of fluid balance charts with either a simple registration of oral fluid intake or with an extended registration, monitoring both the fluid intake (i.e., oral intake, intravenous or intra‐muscular injection and through a feeding tube) and the fluid output (i.e., urine, perspiration, faeces and vomit). In both cases, the registration of the oral fluid intake is done by recording the amount of fluid given to the patient and subtracting the amount of the remaining fluid (Tattersall, 2016). Seemingly, it is a simple procedure; however, in practice, recording fluid intake is a repetitive procedure that requires visual assessment and manual data recording, which are easily affected by human errors and can lead to inadequate and invalid fluid intake measurements (Bak et al., 2017; Pinnington et al., 2016; Tattersall, 2016).

It is well‐known that traditional fluid balance charts are difficult to rely on in daily practice. Fluid measurements in clinical settings can be influenced by the lack of experience and training of staff and patients. In a pilot study done by Jimoh et al. (2015), a low correlation was found between the nursing staff's records and the actual fluid intake by patients when using a standard fluid intake chart. The same study showed that patients performed better than the staff in completing the fluid intake charts. In another pilot study from an intensive care unit, more than 25% of critically ill patients’ fluid balance measurements revealed a deviation of >500 ml, and 79% of the measurements were inaccurate by more than 50 ml. In the same study, a significant association between the inaccurate fluid balance calculations and the administration of diuretics was found (Diacon & Bell, 2014). Furthermore, a qualitative study based on interviews identified nine recurring themes of missed care, with one of them being fluid intake and output documentation. Here, the factors influencing the documentation included that the patient's tray was collected before the fluid intake was documented and a lack of systematic processes for recording the container's refilling (Kalisch, 2006). In addition, other factors such as missing awareness of the importance of the charts, poorly designed charts, high staff turnover, incorrect identification of patients in need of fluid balance assessment or missing guidelines have been found to influence fluid measurements by nurses (Jeyapala et al., 2015; Jimoh et al., 2015; Liaw & Goh, 2018; Yang et al., 2017).

However, these studies have been mainly looking into the knowledge and training in the practical use of a fluid balance chart as a cause of inaccurate fluid balance measurements. Thus, to further explore as to what might be the background for errors in the registration of fluid intake using standard fluid intake charts, the aim of this study was to determine the accuracy and precision in measuring the fluid intake in a clinical setting by three different groups of people, nurses, patients and healthy persons, having different levels of experience in conducting fluid volume assessments, and comparing the use of different types of fluids and fluid containers.

## 2 METHODS

### 2.1 Study design

This study employed a cross‐sectional design involving participants from three groups; nurses, patients and healthy people.

### 2.2 Participant selection and setting

The study took place at a medium sized hospital in the capital region of Denmark. Twenty‐five participants for each group, that is, nurses, patients and healthy persons, were selected for this study. The participants were sampled randomly, as they responded to a general invitation to participate. To ensure a wide range of participants, so that findings were not limited by diagnosis, the invitation was sent out to several departments at the hospital (including gastroenterology, pulmonology, plastic surgery, cardiology, neurology and oncology). Inclusion criteria for all participants were that they should be above the age of 18 years, speak and understand Danish, be cognitively able to give consent and have an absence of tremors.

An additional inclusion criterion for the nurse group participants was that they should have more than 6 months of professional experience as a nurse. For the patient group participants, an additional inclusion criterion was that they should not be diagnosed with severe heart or kidney disease since these patients are well‐trained in the assessment of fluid intake due to the fluid intake restrictions being a part of their medical treatment.

### 2.3 Data collection

**FIGURE 1:**
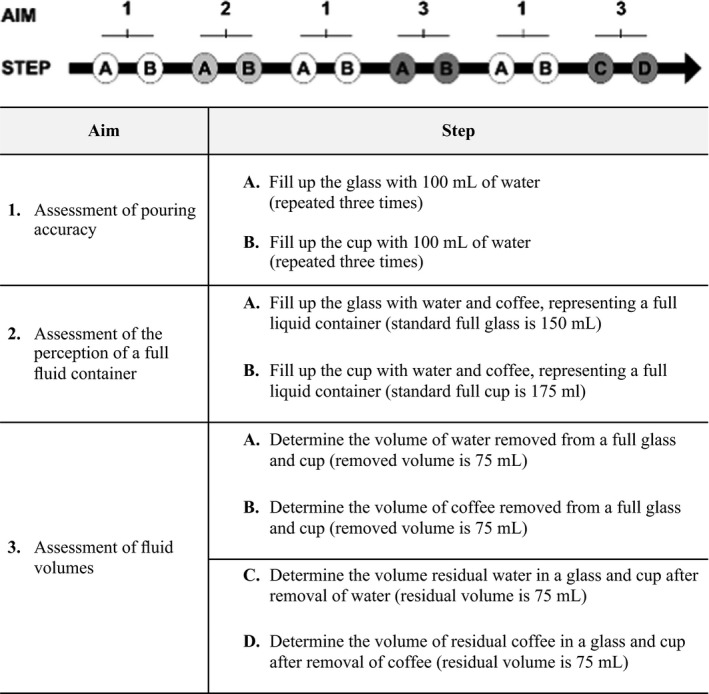
Study framework

**FIGURE 2:**
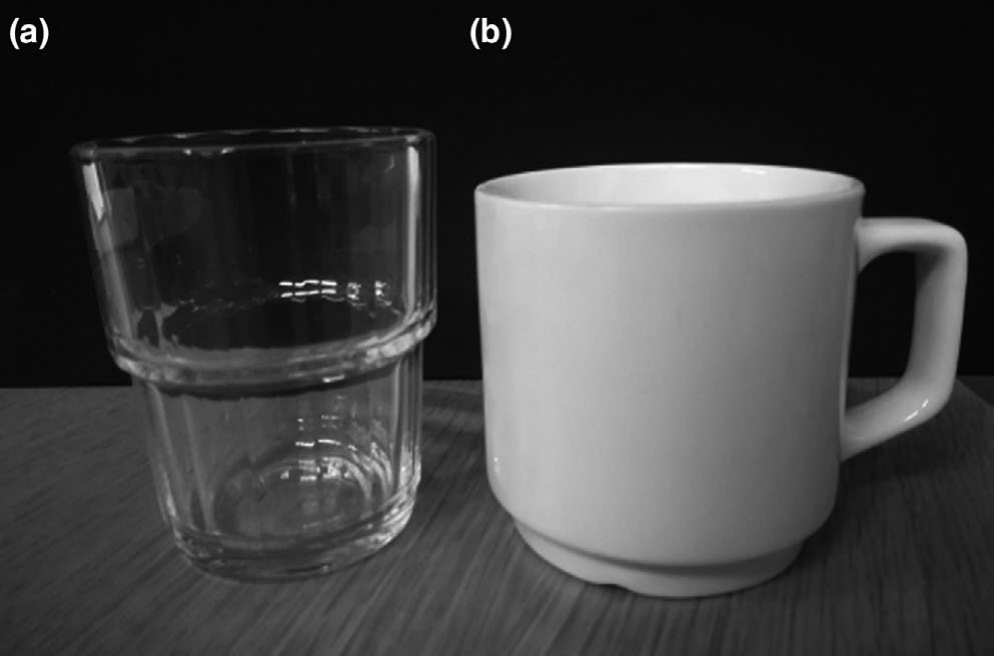
Two standard containers, clear glass (a) and porcelain cup (b), used for the fluid volume assessment

The study was conducted from August 2020 to September 2020. All participants carried out a set of different visual assessments of fluid volume as described in the framework showed in Figure 1. These assessments were carried out using two types of fluids, water (see‐through fluid) and coffee (opaque fluid), which was filled in or removed from two different standard fluid containers used at the hospital, a clear glass (150 ml volume) and a porcelain cup (175 ml volume), which is showed in Figure 2. The two fluid containers were well‐known among the nurses and patients, but not the healthy persons. Each assessment included the use of both fluid containers. Assessment 1A and B was repeated three times by all groups (Figure 1). Immediately after the participants' visual assessments, the exact fluid volumes were measured by a research assistant using a digital scale (KDP 10K‐3, Kern & Sohn GmbH). The scale was an industrial scale, with a readability and repeatability of 1g and provided highly reliable data.

All data were recorded in a spreadsheet (MS Excel, version 16.15, Microsoft Corp.). In addition to the fluid assessments, the participants were asked questions about their daily fluid intake, the type of fluid they preferred to drink, whether see‐through or opaque, the number of hospitalizations they previously went through and their experience with assessing fluid intake volumes.

### 2.4 Data analysis and statistics

The data collected were analysed in an Excel spreadsheet (MS Excel, version 16.15, Microsoft Corp.) using descriptive statistics (numbers and percent), and for continuous data, the mean and standard deviation (*SD*) were included. An acceptable error margin of 10% was set for the accurate evaluation of data, as described in the study by Tattersall (2016). Statistical analysis was carried out using Python (Python Software Foundation, version 3.7).

### 2.5 Ethical consideration

All participants were orally informed about the project. Patients were also given information in writing and had to provide signed consent. All participants were informed about voluntary participation and anonymity, and patients were assured that their participation or rejection to participation would not influence the care they received while being hospitalized. No formal ethical approval was needed according to the Danish law. Data were stored in a secure file, to which only the researchers participating in the study had access. Permission to store data was provided by the Danish Data Protection Agency (nr P‐2020‐821).

## 3 RESULTS

### 3.1 Characteristics of the participant groups

The characteristics for the three groups of participants, nurses, patients and healthy persons, are summarized in Table 1. A higher mean (*SD*) age was recorded for the participants in the patient group, which showed a mean of 54 (13) years, compared to the participants in the nurse group with a mean of 37 (9) years and the healthy group with a mean of 45 (13) years (Table 1). Between the patient and nurse group participants, a similar estimate in the number of full glasses/cups of fluid consumed per day was recorded, with a mean (*SD*) of 11 (3) (Table 1). The estimate for the healthy group participants showed was slightly lower, with a mean (*SD*) of 9 (2) full glasses/cups of fluid consumed per day. The primary fluid consumed was recorded as ‘see‐through’ for all three groups.

**TABLE 1:** Participant group characteristics

Characteristic	Patient (*N* = 25)	Nurse (*N* = 25)	Healthy (*N* = 25)
Age (years), Means (*SD*)	54 (13)	37 (9)	45 (13)
Gender (Female), *N* (%)	14 (56)	22 (88)	12 (48)
Number of hospitalizations, *N* (%)			
≤5 times	20 (80)	N/A	N/A
>5 times	5 (20)		
Experience as a nurse, *N* (%)			
≤10 years	N/A	13 (52)	N/A
>10 years		12 (48)	
Fluid consumed per day (glass/cup), Means (*SD*)	11 (3)	11 (3)	9 (2)
Previous experience with assessing fluid intake, *N* (%)	3 (12)	23 (92)	0 (0)
Colour of fluid most often consumed, *N* (%)			
See‐through	18 (72)	15 (60)	17 (68)

Abbreviations: *N*, number; N/A, not applicable; *SD*, standard deviation.

When asked about their experience in assessing fluid intake, the majority, 92%, of nurse group participants had previously assessed fluid intake. In contrast, only a few, 12%, of the patient group participants had previous experience, and the healthy group participants had no experience.

### 3.2 Pouring accuracy of fluid volume

**FIGURE 3:**
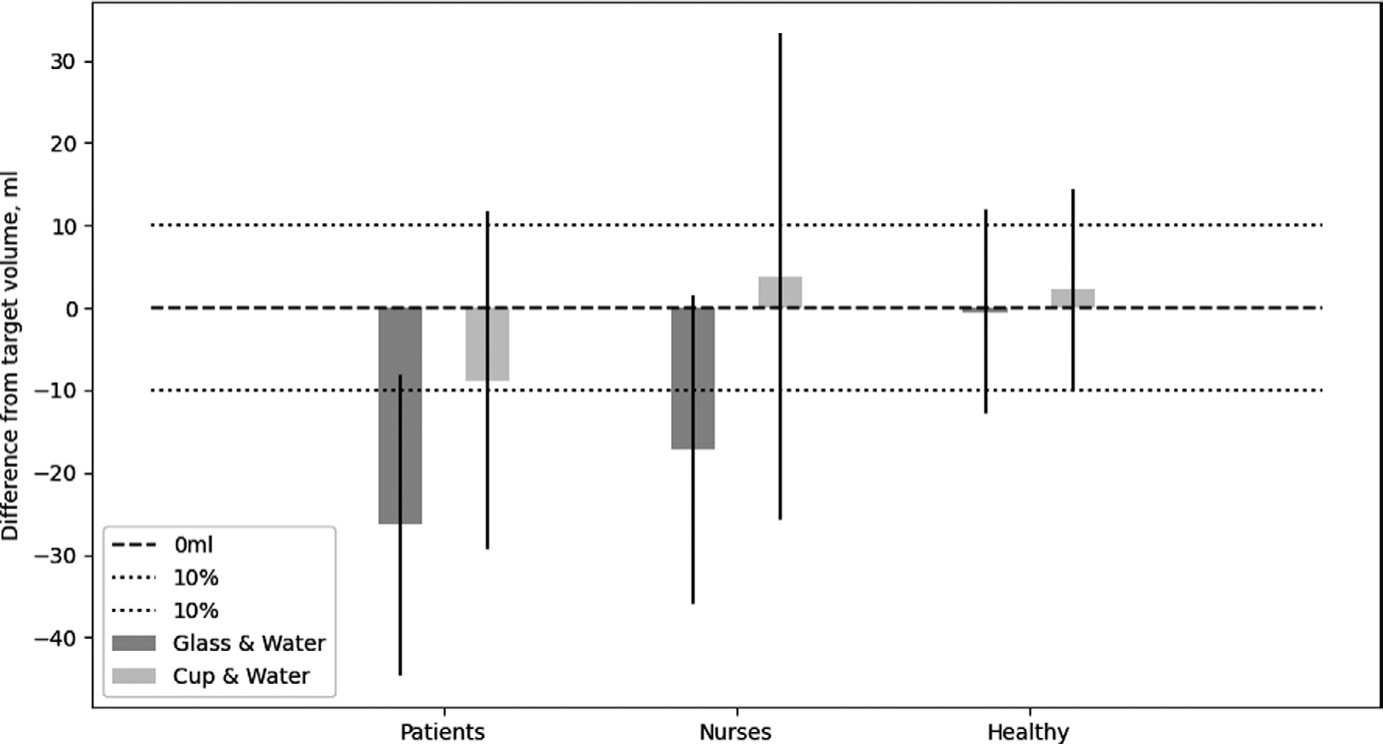
Bar chart showing the mean difference in fluid volumes and standard deviations (SDs) from the target volume of 100 ml (black dashed line, 0 ml), poured in each fluid container, glass (dark grey bars) or cup (light grey bars), for each participant group (patients, nurses, or healthy person). The acceptable 10% error margins (±10 ml) are shown as light grey dashed lines

The participants were asked to repeatedly pour in the same volume of water to a target volume of 100 ml in the two different fluid containers, a glass and a cup. This assessment was repeated three times. The mean volumes of water poured into the fluid containers for each participant group are shown in Figure 3. High variations were observed among the volumes poured in the fluid containers for all participant groups, especially among the patient and nurse groups. The patient group participants poured under the target volume of 100 ml in both the glass and cup, showing a mean difference of −26 and −9 ml, respectively (Figure 3). Furthermore, they showed a high variation (*SD*) in pouring accuracy ranging from 16.9–18.5 ml. The nurse group participants poured under the 100 ml target in the glass, with a mean difference of −17 ml, and poured slightly over the 100 ml target in the cup, with a mean difference of +4 ml.

Interestingly, a very high variation (*SD*) of 27.1 ml was observed in the pouring accuracy for the nurse group participants when they used a cup. In contrast, the variation (*SD*) when using the glass was 17 ml. The healthy group participants poured to target in the glass and close to target in the cup, with a mean difference of −0.5 and +2.2 ml, respectively. Besides, a lower variation (*SD*) in pouring accuracy ranging from 10.5 and 8.2 ml was observed for the healthy group participants (Figure 3).

All three groups showed mean fluid volumes poured in the cups within the acceptable 10% error margin. In contrast, only the healthy group participants showed an acceptable mean volume poured in the glass (Figure 3).

### 3.3 Perception of fluid volumes corresponding to a full fluid container

**FIGURE 4:**
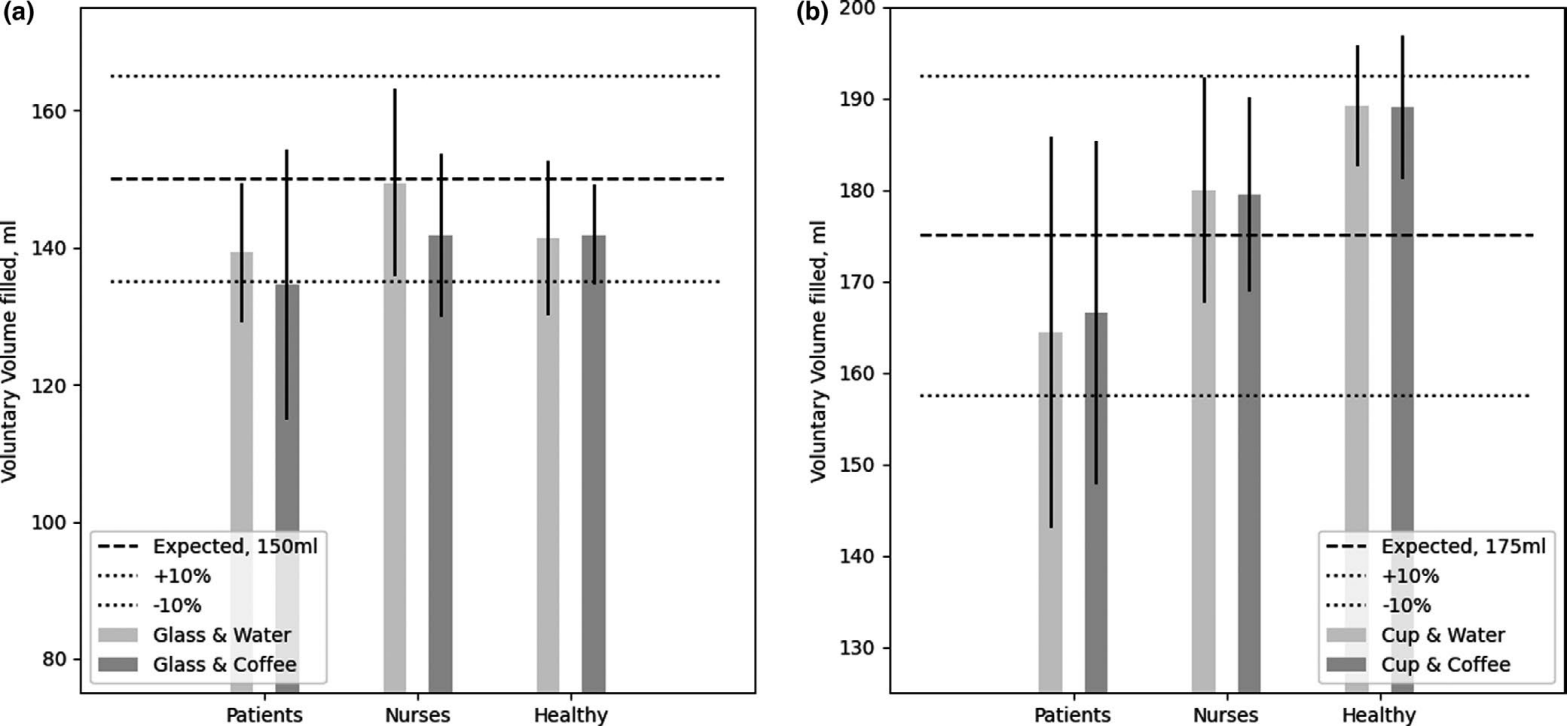
Bar chart showing the mean volumes and standard deviations (SDs) representing the perception of a full fluid container, glass (a) and cup (b), for each fluid type, water (light grey) or coffee (dark grey), and for each participant group (patients, nurses, or healthy person). The expected volumes of the full fluid containers, 150 ml for the glass (a) and 175 ml for the cup (b) are shown as black dashed lines. The acceptable 10% error margins are shown as light grey dashed lines

The participants were asked to fill up each of the two different fluid containers, which were a glass and a cup, with the volume of water or coffee they perceived as a full fluid container. The mean volumes of fluid poured in the two containers by each participant group are shown in Figure 4. At the given hospital setting, a standard full fluid container corresponds to a volume of 150 ml for the glass and 175 ml for the cup. This information was given to all the participants before the assessments.

For all three groups, high variations (*SD*) were observed among the volumes poured in the fluid containers for all participants, especially for the patient and nurse groups. The nurse group participants poured in a mean volume of water in both fluid containers that was closest to the hospital's standard definition of full fluid containers (Figure 4a,b). When coffee was used, the nurse group participants poured a mean volume that was slightly less than a full glass's standard volume. However, the mean volume was still within the acceptable 10% error margin (Figure 4a). A variation (*SD*) of 10.7–13.6 ml was found for this group of participants.

The patient group participants poured mean volumes under the volume of a full fluid container for both fluid types and in both fluid containers. This group of participants showed the highest variation (*SD*) of 10.2–21.4 ml. In contrast, the healthy group participants poured mean volumes under the standard volume of a full glass for both fluid types and poured mean volumes over the standard volume of a full cup for both fluid types. This group of participants showed the lowest variation (*SD*) of 6.6–11.3 ml. Except for the mean volume of coffee poured in the glass by the patient group participants, the mean volumes poured by the patient and healthy group participants were within the acceptable 10% error margin (Figure 4a,b).

### 3.4 Estimates of fluid volumes removed/left in fluid containers

The participants were asked to estimate the fluid volumes that were left in or removed from the two different fluid containers. Both water and coffee were used for the assessments, and the target fluid volume left in/removed was 75 ml in all cases. This information was not given to the participants prior to the assessments.

All the participant groups showed mean estimates above the target volume of 75 ml in all assessments (Table 2). A very high variation (SDs of 31.7–73.1 ml) in the estimates was observed between the participants in the patient group for all fluid types and fluid container types assessed, compared to the two other participant groups, with SDs of 19–28.6 ml for the nurse group and SDs of 12.5–24.8 ml (Table 2). Only the nurse group participants showed mean estimates within the acceptable error margin of 10%, but only for 50% of the assessment cases, which interestingly covered the estimates of removed fluid volumes from the glasses and the estimates of fluid volumes left in the cups (Table 2). The error margin had to be increased to 25% to include all mean estimates of fluid volumes by the nurse group participants (Table 3). Within the 25% error margin, the majority (75%) of the mean estimates of fluid volumes from the healthy group participants were also included. In contrast, none of the mean estimates for this group were included using the 10% error margin (Table 3). None of the mean estimates from the patient group participants were included when the 10% or 25% error margin was used (Tables 2 and 3).

**TABLE 2:** Mean volume estimates of fluid removed (R) from or residual in (I) a container by participant groups

Container	Fluid	Residual in (I)/Removed (R)	Mean estimate, ml (*SD*)		
			Patients	Nurses	Healthy
Glass	Water	75 ml (I)	110.0 (36.3)	89.6 (20.1)	92.4 (17.3)
		75 ml (R)	96.4 (73.1)	79.0 (19.0)	85.4 (14.0)
	Coffee	75 ml (I)	109.2 (38.3)	87.4 (21.8)	86.0 (16.6)
		75 ml (R)	102.6 (58.2)	77.8 (22.0)	86.0 (14.9)
Cup	Water	75 ml (I)	103.8 (45.9)	81.2 (20.8)	88.0 (19.3)
		75 ml (R)	126.4 (64.0)	88.8 (28.6)	101.4 (24.8)
	Coffee	75 ml (I)	101.2 (31.7)	78.2 (22.5)	87.6 (12.5)
		75 ml (R)	127.8 (62.4)	93.8 (27.5)	110.0 (20.0)

Note

Shaded cells are mean volume estimates within the 10% error margin.

**TABLE 3:** Mean volume estimates by participant groups within an acceptable 10% or 25% error margin

Participant group	Mean estimate (%)	
	10% error margin	25% error margin
Patients	0	0
Nurses	50	100
Healthy	0	75

## 4 DISCUSSION

This study explored the different aspects of visual assessments of fluid volumes reflecting the manual monitoring process in clinical practice using fluid balance charts, namely the poring accuracy, the perception of what constitutes a full fluid container and the perception of volumes removed from or left in fluid containers. Overall, a considerable variation in the results was observed in the different assessments for all three participant groups, nurses, patients and healthy persons, as revealed by the high standard deviations. The participants from the nurse and healthy groups were able to more accurately pour an estimated fluid volume of 100 ml compared to the patient group participants when the mean volumes were assessed; however, only the healthy group participants were within the 10% error margin. Previous studies have shown similar results. A study by Tattersall (2016) found that when an error margin of 10% was implemented, only 25% of all the nurses and care staff assessed in a clinical setting provided an acceptable estimate of the volumes of residual fluid in fluid containers. Studies exploring the factors related to unreliable data on patients’ fluid intake have so far mostly been looking into the knowledge and training in the practical use of a fluid balance chart (Jeyapala et al., 2015; Jimoh et al., 2015; Liaw & Goh, 2018; Yang et al., 2017). To our knowledge, only a few studies have been conducted to assess different groups of individuals in a clinical setting to visually estimate volumes of different types of fluids in different standard fluid containers.

The results in this study showed an effect of the fluid container type on the pouring accuracy of fluid volumes. The patient and nurse group participants repeatedly underestimated the volume of water they poured in the container when a glass was used. Also, a high variation in pouring accuracy was seen for the nurse group participants when a cup was used, showing a standard deviation of 27 ml. These findings are consistent with previous studies of various environmental effects on volume perception, which show that different shapes and sizes of fluid containers can influence the pouring accuracy of fluids (Chen & Lee, 2019; Troy et al., 2018). The study by Chen and Lee (2019) further highlighted that the colour of fluid also can affect the volume perception in fluid containers, although this effect was only observed for some of the test groups.

The high variations seen in the volume estimates in this study, both between the groups and within the groups for most assessments, indicates that several factors affected the volume perception by the participants. Another reason for the observed high variations could be that there was a certain amount of calculation involved. The participants, for example, had to estimate the removed volume of fluids in the different fluid containers. Thus, they had to subtract a volume from each container's total volume to ascertain what had been removed, rather than merely stating the volume that was left. In this case, the nurse group participants were able to make more accurate volume estimations (50% of estimations within the 10% error margin) than the two other participant groups. However, further studies are needed to determine whether this was related to the nurses having more experience in assessing volumes of different types of fluids and in different fluid containers, through the use of fluid balance charts, than the patient and healthy group participants.

The clinical implications of unreliable fluid volume assessments might vary from patient to patient, ranging from having no effect to a more severe impact on the effect of treatment and the course of illness (Diacon & Bell, 2014; El‐Sharkawy et al., 2015). This is not only the case for fluid balance monitoring but also for visual assessments of other fluids such as blood loss measurements (Al Kadri et al., 2011) or of patient's intake of food during measurements of dietary intake (Chen & Lee, 2019; Kawasaki et al., 2016). A 10% margin was applied in this study as an acceptable measurement error, as indicated by other studies (Tattersall, 2016). The evidence behind this margin is not well‐established. It might vary between clinical contexts and patients depending on the health situation, which calls for fluid intake surveillance. This study further showed that environmental factors, such as the type of container and the colour of the fluid assessed could lead to a misjudgement in the perceived volume of fluid in the containers, and also that all three participant groups had different perceptions of what volume constituted a full container, which therefore could affect the overall monitoring of daily fluid intake and hydration level. In clinical practice, or when fluid assessment is necessary in patients’ homes, many different containers and types of fluids can be used, which may complicate fluid volume assessments even more compared to the assessments done in this study. More research is therefore needed to investigate critical factors affecting fluid volume assessments in different clinical and healthcare settings to increase patient safety.

### 4.1 Limitations

Despite the inclusion criteria used for including participants in this study, the participating patients might have been in a health‐related condition impacting their ability to assess fluid volumes. All were cognitively able to provide consent but might still have had pain, anxiety or other conditions that affected their overall ability for making such measurements. Most of the nurses had prior experience in fluid volume assessment, whereas most of the patients and healthy persons did not. It is unknown how this experience or the lack of it has impacted the participant's ability to assess volumes. Therefore, specific training might result in more accurate assessments, as seen in some of the results among nurses in this study. In addition, the use of standard hospital fluid containers in this study might have given nurses and patients the advantage of having seen these containers before, whereas the healthy persons might not have seen them before. Even so, both the glass and the cup used in this study did however reflect the size of other cups and glasses often used in a Danish context.

## 5 CONCLUSION

The results in this study point to several potential areas in which future solutions towards more accurate fluid volume measurements can be developed depending on the context and purpose of the monitoring process. In addition, more focus on accurate monitoring of fluid balance and oral fluid intake might include new ways of collaboration and sharing of data across healthcare sectors and medical‐technical equipment, thereby improving patient care, increasing patient safety and preventing illness. This will also enable nurses to link fluid intake to patient care outcomes and support them in their clinical decision‐making. Low technical solutions could include using glasses or cups with a printed measure of volume on the inside or outside. High technical solutions could include hydration monitoring apps (Steven et al., 2019) or, for example, smart water bottles that can do standard volume measurements and detect changes in contained volumes (Borofsky et al., 2018).

## CONFLICT OF INTEREST

MBSS and MLB are affiliated with an organization with a non‐financial interest in the subject matter discussed in this manuscript.

## AUTHOR CONTRIBUTIONS

CFM, MBSS, MLB and HK designed and directed the project; HK collected the data; CFM analysed the data; CFM and HK wrote the article. All authors discussed the results and commented on the manuscript.

## Data Availability

The data that support the findings of this study are available from the corresponding author upon reasonable request.
